# Evaluation of a Smoking Cessation Program for HIV Infected Individuals in an Urban HIV Clinic: Challenges and Lessons Learned

**DOI:** 10.1155/2014/237834

**Published:** 2014-10-01

**Authors:** Debra Chew, Michael B. Steinberg, Polly Thomas, Shobha Swaminathan, Sally L. Hodder

**Affiliations:** ^1^Department of Medicine, Division of Infectious Diseases, Rutgers New Jersey Medical School, Newark, NJ, USA; ^2^Division of Infectious Diseases, Rutgers New Jersey Medical School, Ambulatory Care Center-Infectious Disease Practice, 140 Bergen Street, Newark, NJ 07103, USA; ^3^Division of General Internal Medicine, Rutgers Robert Wood Johnson Medical School, New Brunswick, NJ, USA; ^4^Department of Preventive Medicine and Community Health, Rutgers New Jersey Medical School, Newark, NJ, USA

## Abstract

*Introduction.* HIV infected persons have high prevalence of smoking and tobacco-associated health risks. Few studies describe smoking cessation programs targeting this population. The Infectious Disease Practice (IDP) in Newark, New Jersey, initiated a smoking cessation program (SCP) for HIV infected smokers. We report participation, abstinence rates, and predictors of abstinence. *Methods.* This is a prospective cohort study, comparing participants to non-SCP smokers, during April 1, 2011, to October 31, 2012. Intervention included one individualized counseling session with an offer of pharmacotherapy. Univariate and multivariate analyses were performed with self-reported seven-day point prevalence abstinence at six months as primary outcome measure. *Results.* Among 1545 IDP patients, 774 (51%) were current smokers of whom 123 (16%) participated in the SCP. Mean six-month abstinence rate amongst SCP participants was 16%. A history of cocaine or heroin use was predictive of continued smoking (odds ratio [OR] adjusted 0.20, 95% confidence interval [CI] 0.07–0.55) while smokers in the preparation stage of change were more likely abstinent at six months (OR adjusted 8.26, 95% CI 1.02–66.67). *Conclusions.* A low-intensity smoking cessation intervention in an HIV treatment setting is effective in a minority of participants. Further research is needed to better address barriers to smoking cessation such as substance use.

## 1. Introduction

Smoking is highly prevalent among persons living with HIV and is associated with a high risk for tobacco-associated morbidity and mortality. More than half of the persons living with HIV in the United States are current smokers, with estimated smoking rates ranging from 50 to 70% compared to 20% in the general population [1–8].

The high prevalence of smoking has profound health implications for HIV infected individuals, with smoking linked to a myriad of health risks and increased mortality in this population [2, 4, 9]. Lifson et al. [9] estimated that 24% of deaths among persons with HIV in the Strategies for Management of Antiretroviral Therapy Clinical Trial (SMART) were attributable to smoking. Cardiovascular disease accounts for a large majority of deaths, and smoking is a significant cofactor in the premature development of cardiovascular disease. HIV infected smokers compared with nonsmokers have increased risk for myocardial infarction [9–11] and greater rates of chronic obstructive pulmonary disease [12, 13], non-AIDS related malignancies, [9, 14–16], and both HIV and non-HIV related infectious complications, including oral candidiasis and bacterial pneumonia [2, 9, 17–19]. Additionally, there are conflicting data whether cigarette smoking adversely affects immunologic response to HAART [6]. Finally, studies have shown that HIV infected smokers experience decreased quality of life [3, 20, 21].

Despite the critical need to deliver smoking cessation interventions to HIV infected smokers, there are few published studies on tobacco cessation interventions targeting this group. There are neither formal guidelines for tobacco dependence treatment nor recommendations on smoking cessation programs tailored for HIV infected persons. To date, there have been only several small studies and a few randomized controlled trials (RCTs) employing individualized behavioral strategies (either face-to-face, telephone, cellphone, or computer-based delivered counseling) with nicotine replacement therapy (NRT) [22–27], tailored group support with NRT [28, 29], or single arm pharmacotherapy trials with bupropion or varenicline [30, 31]. Moreover few studies have tailored their smoking cessation interventions to be incorporated into routine HIV clinical care and delivered with a treatment intensity that is feasible in established HIV clinical settings.

Given the scant data on tobacco dependence treatment for HIV infected smokers and high prevalence of smoking among our patient population, we initiated a smoking cessation program at the Infectious Disease Practice (IDP), an inner-city HIV care clinic in Newark, New Jersey. Our current study evaluates the effectiveness of the IDP smoking cessation program (SCP) for HIV infected smokers with the aim of informing on future effective tobacco cessation interventions for HIV infected smokers in an HIV treatment setting.

## 2. Materials and Methods

We conducted a prospective cohort study of SCP participants with a case-control analysis comparing SCP participants with non-SCP participants. The study period was April 1, 2011 to October 31, 2012. Current smokers were recruited to the IDP tobacco cessation program between April 1, 2011, and May 31, 2012. Participants in the smoking cessation program were subsequently followed for 6 months for self-reported tobacco abstinence, the primary outcome. The cohort of all IDP clients during the study period was ascertained retrospectively via the IDP Ryan White Champs database and SCP non-participants were compared to SCP participants. Our objectives were to determine program participation rates, abstinence rates, and evaluate predictors of abstinence. Our study protocol is approved by Rutgers Newark, New Jersey Institutional Review Board.

### 2.1. Study Setting and Study Population

The Infectious Disease Practice provides comprehensive HIV-primary care to approximately 1,500 adult HIV infected patients in the greater Newark area, the majority of whom are minorities of low socioeconomic status. Patients were eligible for this study if they had confirmed HIV infection (by HIV Western blot), had an IDP medical visit between April 1, 2011 and April 30, 2012, reported current smoking at that visit (smoked any tobacco in the previous 7 days), and were interested in quitting smoking.

### 2.2. Smoking Cessation Program Intervention and Recruitment

Beginning in April 2011, we recruited eligible subjects into the IDP smoking cessation program. All IDP patients who smoked were offered to participate in the program. Patients were referred by their clinic providers, clinic staff, or self-referred via program fliers in the waiting room or exam rooms. Patients' smoking status was routinely assessed at each clinic visit, and providers individually assessed clients for their willingness to quit tobacco. Patients who agreed to participate in the smoking cessation program received at least a single individualized face-to-face counseling session at the time of enrollment that included assessment of tobacco use and behaviors. Counselors (either a physician, case manager, peer navigator, or mental health counselor all trained in tobacco cessation) assisted participants with a quit plan and provided practical problem solving/skills training with quitting smoking (e.g., review of triggers, withdrawal symptoms, coping strategies), and provided support. The counseling session was usually about 1 hour long, and delivered on-site by counselors trained in smoking cessation. Tobacco cessation pharmacotherapy was either prescribed at the initial counseling visit or on follow-up visit with the physician directing the program. Participants were followed for tobacco abstinence with scheduled program follow-up visits, or telephone calls, or by routine medical clinic visits to their providers. Follow-up visits assessed smoking status, adherence to treatment, and barriers to quitting. Incentives were not available for program participants.

### 2.3. Measures

The primary outcome measure of this study was tobacco abstinence at 6 months. A secondary outcome measure was participation in the tobacco cessation program among current smokers. Tobacco abstinence was assessed via self-reported, 7 day point prevalence (no tobacco smoking in the prior 7 days). Current smoking status was assessed at each clinical visit; beginning in March 2012, this was systematically documented in the IDP Ryan White Champs database. Current smoking was defined as having smoked any tobacco in the previous 7 days.

Other independent variables collected and analyzed included socio-demographic factors, HIV-related factors, medical history, tobacco use and behaviors, history of mental illness, current and past substance use, and program factors. Virologic suppression was defined as having last HIV viral load less than 400 copies/mL on HAART. Tobacco use and behaviors were assessed via a baseline questionnaire given to smoking cessation program participants. Nicotine dependence was assessed with the Modified Fagerstrom Test for Nicotine Dependence Questionnaire (FTND) [32]. The Smoking Stage of Change (short form) [33] was used to assess the participant's readiness to quit at baseline. Belief in the importance of quitting and confidence in the ability to quit smoking were measured using a 1–10 point visual analogue scale on which participants rated their importance or confidence. Program factors included smoking cessation counselor, referral source, adherence with initial pharmacotherapy visit, pharmacotherapy prescribed and type, insurance reimbursement for pharmacotherapy (by self-report), and self-reported adherence to pharmacotherapy at each follow-up.

### 2.4. Data Collection

Data was collected via medical chart review and interviewer-administered patient questionnaire on initial tobacco cessation program entry. All SCP participants were asked to complete an initial interviewer-administered patient questionnaire, administered at the time of study enrollment that ascertained baseline tobacco, medical, and psycho-social history. Medical charts were reviewed to confirm smoking status, medical history, history of mental illness and substance use, and follow-up smoking status, pharmacotherapy treatment and insurance reimbursement and adherence to pharmacotherapy. Current smoking status and date, demographic data, HIV risk factor, receipt of HAART, last HIV viral load and Cd4 cell count were electronically abstracted from medical charts.

### 2.5. Statistical Analyses

Descriptive analyses were generated to characterize the SCP and overall clinic cohorts. Dichotomous variables were analyzed using Chi square or Fisher's exact test. Comparison of means was accomplished using Student *t*-test or Kruskall-Wallis *H* test. A series of chi-square or *t*-test analyses were performed to test the association of 6 month tobacco abstinence (the primary outcome of interest) with other patient demographic, tobacco, medical, HIV, psychosocial, and program related factors among SCP participants; and the association of SCP participation (secondary outcome measure) with demographic, HIV, and clinical factors among current smokers in the IDP clinic. Participants who did not complete the 6-month follow-up were assumed to be smoking and coded as non-abstinent. Additionally, participants who did not complete their 6 month follow-up, but who later reported smoking on subsequent follow-up visits, were also classified as non-abstinent. A backward, stepwise multiple logistic regression model was generated to identify predictors of tobacco abstinence by comparing abstinence among program participants while controlling for other independent variables. The following continuous variables were dichotomized in multivariate analyses with nicotine dependence categorized by moderate to high dependence (FTND score ≥5) versus low dependence (FTND score <5), and confidence in quitting smoking (>7 versus ≤7). The multivariate model included demographic variables and variables that were associated (*P* ≤ 0.10) with tobacco abstinence on univariate analysis. Odds ratios with corresponding 95% Confidence intervals were generated to estimate strength of the association. Data analyses were performed using with SAS version 9.13.

## 3. Results

A total of 1545 patients had one or more IDP clinic visit during the study period. Of these, 774 (51%) were current smokers, 262 (17%) were former smokers, and 481 (32%) were never smokers; 28 patients had unknown smoking status. Of current smokers, 123 (16%) agreed to participate in the smoking cessation program.

Table 1 shows characteristics of the SCP participants and smokers who were non-SCP participants. Mean age of SCP participants was 50, 53% of SCP participants were male, 85% were black, and 29% were uninsured. Compared to current smokers who did not participate in the smoking cessation program, participants were significantly older and insured. SCP participants also had more controlled HIV disease reflected by significantly higher CD4 cell count (539 versus 429 cells/*μ*L) and greater virologic suppression on HAART (78% versus 62%, although not statistically significant) than smokers who did not participate in the smoking cessation program.

Co-morbid medical and psychiatric disorders and substance use were highly prevalent among participants. Among SCP participants, 42% had hypertension, 12% heart disease, 26% asthma or COPD, 11% diabetes and 25% hepatitis C co-infection (not shown in table). About half of participants (52%) had a history of mental illness (43% depression, 22% anxiety, 15% bipolar disease and 6% schizophrenia). While current drug use was low, many reported a history of past substance use (41% alcohol, 27% marijuana, 58% cocaine use, and 41% heroin use).

On average, participants smoked 11.0 cigarettes/day and had 2.1 prior quit attempts. Forty-three percent reported living in a household with another smoker. The majority had moderate nicotine dependence with a mean 4.6 FNTD. Most were ready to quit by stage of change: 72% in preparation stage, 24% in contemplation stage, and only 4% in precontemplation stage.

Eighty-two percent of SCP participants were prescribed pharmacotherapy for tobacco dependence (96% with NRT, 5% varenicline, 16% buproprion, and 54% received combination treatment). Of those treated with pharmacotherapy, most (83%) were not adherent to cessation treatment on at least one or more followup visit. Nearly half (48%) reported that their cessation treatment was not fully reimbursed.

The six-month abstinence rate among SCP participants was 16%. Factors associated with abstinence in univariate analysis are shown in Table 2 and included lower tobacco dependence by the FTND and higher readiness to quit by stage of change. Current or past cocaine or heroin use was associated with continued smoking among SCP participants.

Predictors of six-month abstinence are shown in Table 3. In a final multivariate model that included age, sex, race, history of mental illness, past or current cocaine or heroin use, nicotine dependence, and stage of change, a history of cocaine or heroin use was significantly associated with continued smoking, while stage of change was significantly associated with tobacco abstinence. Participants with a history of illicit cocaine or heroin use were 5 times less likely to be abstinent than those without illicit drug use. Participants who were in the preparation stage of change were 8 times more likely to be abstinent than those either in the precontemplation or contemplation stage. Other factors including demographic factors, history of mental illness, and nicotine dependence were not significant predictors of tobacco abstinence.

## 4. Discussion

In summary, we found that the IDP smoking cessation program intervention was associated with an overall 6-month abstinence rate of 16%, comparable to other published trials among HIV infected smokers in various treatment settings. We found that patients who had a history of cocaine or heroin use and those who were less motivated to quit were less likely to be abstinent. We also found the intervention to be limited by a low SCP participation (16%) despite an overall high clinic smoking prevalence (51%).

Our tobacco abstinence outcome is similar to that found in other published trials that employed more intensive individualized and group counseling interventions along with NRT among HIV infected smokers. A cessation rate of 16% compares with other RCTs demonstrating a 3–6-month abstinence rate ranging from 9 to 37% [23–25, 29]. Our abstinence rate also is similar to the mean 16.8% derived from meta-analysis of individualized counseling interventions conducted in other non-HIV populations [34].

While our abstinent rate is encouraging, it also demonstrates the challenges to treating tobacco dependence in this population. As a group, HIV infected smokers have a multitude of complex and interactive social, economic, psychiatric, and medical needs that may represent serious obstacles to effective cessation efforts. Our cohort is typical of an inner-city HIV-care clinic population in the United States, which is predominantly of ethnic minority groups and low socioeconomic status. Comorbid psychiatric disorders and illicit substance use, as demonstrated in our cohort, are highly prevalent, where approximately half had a history of mental illness, alcohol use, and cocaine or heroin use. A similarly high prevalence of mental illness and substance abuse have also been reported among HIV infected smokers in other cohorts [1, 5, 7, 35, 36], all of which are associated with high risk for smoking and reduced success for abstinence [34].

In this study, we identified a history of illicit cocaine or heroin use to be a major predictor of nonabstinence. This finding is not surprising given that substance use is a well-recognized barrier to successful cessation [34]. Nicotine and other drugs of abuse have a shared neurobiological link, targeting the dopaminergic reward pathway. Nicotinic receptor activation is an important mediator in sensitization to stimulants like cocaine [37]. Additionally, smoking and drug use share similar psychological and physical cues and withdrawal symptoms, which may reinforce addictive behaviors and lead to difficulties in quitting smoking [37, 38]. Our finding suggests that effective smoking cessation interventions may need to be tailored to address particular needs of HIV infected smokers either with active or prior addiction to illicit drugs and that they may benefit from more intensive cessation approaches or strategies that incorporate substance use counseling and mental health services into cessation interventions.

Not surprisingly we found that smokers who were more ready to quit by stage of change were more likely to be abstinent. Prochaska et al. [39] found that smokers in the preparation stage at baseline consistently reported more cessation than smokers in contemplation who reported more abstinence than smokers in the precontemplation stage. Meta-analyses across various behaviors, including smoking, have confirmed that having an intention to change behavior is critical to promoting change [40]. Current clinical practice guidelines recommend using motivational interviewing techniques to motivate those smokers unwilling to quit by targeting the 5Rs (personally relevant reasons to quit, risks associated with continued smoking, rewards for quitting, roadblocks to successful quitting, and repetition of counseling at subsequent health care visits) [34].

In this study, we found adherence to tobacco cessation pharmacotherapy to be markedly low. Nonadherence is common in those with HIV and continues to be a major challenge in treating the HIV population [41, 42]. Poor adherence to tobacco cessation pharmacotherapy greatly reduces the success of smoking cessation as pharmacotherapy can effectively double tobacco abstinence rates [34]. Among HIV infected smokers, Ingersoll et al. [24] found that only 37% of participants in a small RTC were compliant with the patch at 3 months of followup. This suggests that smokers receiving pharmacotherapy should be closely monitored for compliance and counseled on the importance of adherence to treatment success and the effectiveness of tobacco cessation medications. At the same time, providers should discuss patient concerns and myths regarding treatment [43]. Further research should be done to explore patient barriers to pharmacotherapy compliance including knowledge, attitudes, and perceptions of HIV infected smokers regarding cessation treatment.

Our program was limited by the low participation among clinic smokers. While lack of interest from patients may account for some of the low participation, uneven referral rates from clinic providers also contributed in large part to the low rate of participation despite frequent assessment of smoking during clinic visits. Surveys among HIV care providers have found that the greatest barrier to providing smoking interventions in treatment settings is lack of time and lack of confidence in addressing smoking cessation [44–46]. Shuter et al. [46] found that only 20% of HIV care providers had received prior formal tobacco dependence training. Systemic changes to health care delivery, such as electronic medical record systems, reminder systems, provider and nonclinician training, and clinician feedback, may help improve assessment and treatment of smokers in medical care [34, 43]. Ways to shift responsibility of engaging patients about their smoking behaviors to other nonclinician staff may be investigated as possible solutions to address structural concerns with time constraints during provider visits. Since the HIV care delivery model is based on providing integrated, comprehensive care using a multidisciplinary approach, case managers, substance abuse and mental health counselors, and other HIV clinical staff may help engage and counsel HIV infected patients regarding their tobacco behaviors and address barriers that influence smoking cessation. Providers should at the minimum provide brief advice to quit and offer cessation pharmacotherapy for patients willing to quit at each clinical encounter, as recommended in the U.S. Public Health Service clinical practice guidelines for treating tobacco use and dependence [34].

Additional resources to support tobacco dependence interventions are needed to implement effective smoking cessation interventions for HIV infected smokers. This not only includes resources for providing tailored, more intensive approaches to this population but also includes providing tobacco dependence pharmacotherapy as a covered benefit by health insurance plans. Nearly half of our participants did not receive reimbursement for their pharmacotherapy regimen. Access to effective pharmacotherapy is especially problematic among HIV infected persons as they are disproportionately of low income and often lack health insurance.

There are several major limitations in this study worth noting. The study lacked a comparison measure, as abstinence among non-SCP participants was unknown. Also, as a nonrandomized study, possible biases and confounding may have occurred, including nonparticipation and referral bias. Although virologic suppression on HAART did not predict abstinence, it is likely participants were more motivated to quit smoking and may possibly have had fewer risk factors associated with cessation failure, including substance disease or mental health issues. Our small sample size may have limited our ability to assess associations between a range of psychosocial, clinical, tobacco-related, and program factors and abstinence outcome, and limited our ability to detect less common findings. Additionally, validity and reliability of the abstinence outcome may have been affected as it lacked biochemical confirmation. In the SCP, 18% of participants failed to attend followup pharmacotherapy visit and thus did not receive optimal intervention. Followup of participants ranged greatly among the participants, also leading to varied intervention intensity among participants. Finally, our study was limited to HIV infected smokers who were primarily motivated to quit. Although our clinic population is typical of other urban HIV care clinics in the United States, our findings may not be generalizable to other populations of HIV infected smokers or to those less motivated to quit.

## 5. Conclusions

This study provides evidence that HIV infected smokers can quit smoking with a relatively low-intensity intervention in an HIV treatment setting. Substance use and nonadherence were identified as obstacles to achieving abstinence and suggest that additional or more intensive strategies are needed given the high prevalence of these risk factors among HIV infected smokers. Further studies are needed to determine which strategies are the most effective in treating tobacco dependence in this population, how to best tailor these interventions to address the unique concerns and needs of smokers living with HIV/AIDS, and how to best integrate tobacco dependence treatment into routine HIV care. In the meantime, barriers to delivering effective smoking cessation interventions in the health care system should be addressed. Providers must maximize the opportunity to deliver smoking cessation interventions to their HIV infected patients by recognizing and consistently offering smoking cessation treatment to patients who smoke as outlined in current clinical practice guidelines for treating tobacco use and dependence.

## Figures and Tables

**Table 1:** Characteristics of participants and nonparticipants and factors associated with participation in IDP smoking cessation program among current smokers (*N* = 774).

Variable	Smoking cessation program participant (*N* = 123)		Smoking cessation program nonparticipant (*N* = 651)		*P* value^1^
	Mean (SD)	*n* (%)	Mean (SD)	*n* (%)	
Age	50.0 (9.1)		47.3 (10.0)		**0.01**
Sex					
Male		65 (52.8)		370 (56.8)	0.41
Female		58 (47.2)		281 (43.2)	
Race/Ethnicity					
Black		105 (85.4)		554 (85.1)	0.94
Nonblack		18 (14.6)		97 (14.9)	
Insurance status					
Any		87 (70.7)		391 (60.1)	
None		36 (29.3)		260 (39.9)	**0.03**
Most recent CD4+ count (cells/*μ*L)	538.0 (323)		429 (329)		**<0.01**
Virologic suppression on HAART^2^					
Yes		92 (78.0)		368 (62.0)	0.10
No		26 (22.0)		226 (38.1)	

SD: standard deviation.

^1^By chi-square for categorical variables or Kruskall-Wallis for continuous variables.

^2^Defined by HIV VL <400 copies/mL.

**Table 2:** Factors associated with 6-month tobacco abstinence among IDP smoking cessation program participants (*N* = 123)^1^.

Variable	Abstinent		Nonabstinent		*P*-value^2^
	(*N* = 20)		(*N* = 97)		
	Mean (SD)	*n* (%)	Mean (SD)	*n* (%)	
Demographic factors					
Age	50.2 (11.2)		49.9 (8.7)		0.54
Male sex		10 (50.0)		55 (53.4)	0.78
Black race/ethnicity		17 (85.0)		88 (85.4)	1.0
Insurance status, any		17 (85.0)		70 (68.0)	0.13
HIV-related factors					
CD4, cells/*μ*L^3^	606 (359)		525 (316)		0.33
Virologic suppression on HAART^4^		18 (90.0)		74 (75.5)	0.24
Psychosocial factors					
History of mental illness		7 (35.0)		57 (55.3)	0.10
History of depression		6 (30.0)		47 (45.6)	0.20
History of anxiety		3 (15.0)		24 (23.3)	0.56
Current alcohol use		2 (10.0)		13 (12.6)	1.0
Current marijuana use		2 (10.0)		9 (8.7)	1.0
Current cocaine use		3 (15.0)		6 (5.8)	0.16
Current heroin use		0 (0.0)		4 (3.9)	1.0
Alcohol use, any		9 (45.0)		42 (40.8)	0.75
Marijuana use, any		4 (20.0)		29 (28.2)	0.45
Cocaine use, any		6 (30.0)		65 (63.1)	**0.01**
Heroin use, any		4 (20.0)		47 (45.6)	**0.03**
Tobacco-related factors					
Cigarettes/day	10.5 (6.2)		11.1 (7.6)		0.83
Age started smoking	16.3 (4.2)		16.4 (4.6)		0.93
Prior quit attempts	1.6 (1.3)		2.2 (2.8)		0.35
Mean FTND (SD)^5^	3.8 (1.9)		4.7 (2.2)		**0.05**
Mean importance of quitting smoking (SD)^6^	9.0 (1.5)		9.2 (1.6)		0.68
Mean confidence in quitting smoking (SD)^6^	8.5 (2.0)		7.6 (2.4)		0.11
Other household smokers		6 (30.0)		47 (45.6)	0.20
Preparation stage of change^7^		19 (95.0)		74 (72.6)	**0.04**
Pharmacotherapy prescribed		18 (90.0)		78 (75.7)	0.16

SD: standard deviation; FTND: Fagerstrom Test for Nicotine Dependence.

^1^6 participants who did not complete 6 month f/u were classified as nonabstinent.

^2^By chi-square or 2-sided Fisher's exact for categorical variables and Kruskall-Wallis for continuous variables.

^3^Last documented CD4 cells/*μ*L during study period.

^4^Defined by HIV VL <400 copies/mL on HAART.

^5^As measured by the modified Fagerstrom Test for Nicotine Dependence (FTND); score of ≥5 indicates significant nicotine dependence; score of ≤4 indicates low-moderate nicotine dependence.

^6^0–10 Likert scale belief items with higher numbers indicating more importance or confidence.

^7^As measured by Stage of Change short form, excluding 1 missing value.

**Table 3:** Multivariate analysis of predictors associated with tobacco abstinence^1^.

Variable	OR (95% CI)	*P*-value	OR_Adj_ (95% CI)	Adjusted *P* value
Age < 50, years	0.86 (0.32–2.28)	0.76	NS	
Male sex	0.87 (0.34–2.28)	0.78	NS	
Black race	0.97 (0.25–3.70)	0.96	NS	
History of mental illness	0.44 (0.02–1.18)	0.10	NS	
Cocaine or heroin use, past or current	0.23 (0.08–0.61)	**0.00**	0.20 (0.07–0.56)	**0.002**
Moderate/high nicotine dependence (FTND ≥5)^2^	0.41 (0.15–1.09)	0.07	NS	
Preparation Stage of Change^3^	7.19 (0.92–56.24)	0.06	8.26 (1.02–66.67)	**0.048**

OR = odds ratio; CI = confidence interval; OR_Adj_ = adjusted odds ratio; NS = not significant.

^1^Final model included age, sex, race, history of mental illness, history of heroin or cocaine use, nicotine dependence, and stage of change; *N* = 122 due to 1 missing value.

^2^As measured by the modified Fagerstrom Test for Nicotine Dependence (FTND); score of ≥5 indicates significant nicotine dependence; score of ≤4 indicates low-moderate nicotine dependence.

^3^As measured by stage of change short form: precontemplation and contemplation versus preparation stage of change, excluding 1 missing value.

## References

[1] Burkhalter JE, Springer CM, Chhabra R, Ostroff JS, Rapkin BD (2005). Tobacco use and readiness to quit smoking in low-income HIV-infected persons. *Nicotine and Tobacco Research*.

[2] Burns DN, Hillman D, Neaton JD (1996). Cigarette smoking, bacterial pneumonia, and other clinical outcomes in HIV-1 infection. *Journal of Acquired Immune Deficiency Syndromes and Human Retrovirology*.

[3] Crothers K, Griffith TA, McGinnis KA (2005). The impact of cigarette smoking on mortality, quality of life, and comorbid illness among HIV-positive veterans. *Journal of General Internal Medicine*.

[4] Feldman JG, Minkoff H, Schneider MF (2006). Association of cigarette smoking with HIV prognosis among women in the HAART era: a report from the women's interagency HIV study. *The American Journal of Public Health*.

[5] Gritz ER, Vidrine DJ, Lazev AB, Amick BC, Arduino RC (2004). Smoking behavior in a low-income multiethnic HIV/AIDS population. *Nicotine and Tobacco Research*.

[6] Mamary EM, Bahrs D, Martinez S (2002). Cigarette smoking and the desire to quit among individuals living with HIV. *AIDS Patient Care and STDs*.

[7] Webb MS, Vanable PA, Carey MP, Blair DC (2007). Cigarette smoking among HIV+ men and women: examining health, substance use, and psychosocial correlates across the smoking spectrum. *Journal of Behavioral Medicine*.

[8] Dube SR, McClave A, James C, Caraballo R, Kaufmann R, Pechacek T (2010). Vital signs: current cigarette smoking among adults aged ≥18 years—United States, 2009. *Morbidity & Mortality Weekly Report*.

[9] Lifson AR, Neuhaus J, Arribas JR, van Berg-Wolf MD, Labriola AM, Read TRH (2010). Smoking-related health risks among persons with HIV in the strategies for management of antiretroviral therapy clinical trial. *The American Journal of Public Health*.

[10] Friis-Møller N, Reiss P, Sabin CA (2007). Class of antiretroviral drugs and the risk of myocardial infarction. *New England Journal of Medicine*.

[11] Friis-Møller N, Sabin CA, Weber R (2003). Combination antiretroviral therapy and the risk of myocardial infarction. *The New England Journal of Medicine*.

[12] Crothers K, Butt AA, Gibert CL, Rodriguez-Barradas MC, Crystal S, Justice AC (2006). Increased COPD among HIV-positive compared to HIV-negative veterans. *Chest*.

[13] Diaz PT, King MA, Pacht ER (2000). Increased susceptibility to pulmonary emphysema among HIV-seropositive smokers. *Annals of Internal Medicine*.

[14] Chaturvedi AK, Pfeiffer RM, Chang L, Goedert JJ, Biggar RJ, Engels EA (2007). Elevated risk of lung cancer among people with AIDS. *AIDS*.

[15] Engels EA, Brock MV, Chen J, Hooker CM, Gillison M, Moore RD (2006). Elevated incidence of lung cancer among HIV-infected individuals. *Journal of Clinical Oncology*.

[16] Kirk GD, Merlo C, O’Driscoll P (2007). HIV infection is associated with an increased risk for lung cancer, independent of smoking. *Clinical Infectious Diseases*.

[17] Conley LJ, Bush TJ, Buchbinder SP, Penley KA, Judson FN, Holmberg SD (1996). The association between cigarette smoking and selected HIV-related medical conditions. *AIDS*.

[18] Gordin FM, Roediger MP, Girard P-M (2008). Pneumonia in HIV-infected persons: increased risk with cigarette smoking and treatment interruption. *The American Journal of Respiratory and Critical Care Medicine*.

[19] Miguez-Burbano MJ, Burbano X, Ashkin D (2003). Impact of tobacco use on the development of opportunistic respiratory infections in HIV seropositive patients on antiretroviral therapy. *Addiction Biology*.

[20] Turner J, Page-Shafer K, Chin DP (2001). Adverse impact of cigarette smoking on dimensions of health-related quality of life in persons with HIV infection. *AIDS Patient Care and STDs*.

[21] Vidrine DJ, Arduino RC, Gritz ER (2007). The effects of smoking abstinence on symptom burden and quality of life among persons living with HIV/AIDS. *AIDS Patient Care and STDs*.

[22] Elzi L, Spoerl D, Voggensperger J (2006). A smoking cessation programme in HIV-infected individuals: a pilot study. *Antiviral Therapy*.

[23] Humfleet GL, Hall SM, Delucchi KL, Dilley JW (2013). A randomized clinical trial of smoking cessation treatments provided in HIV clinical care settings. *Nicotine and Tobacco Research*.

[24] Ingersoll KS, Cropsey KL, Heckman CJ (2009). A test of motivational plus nicotine replacement interventions for HIV positive smokers. *AIDS and Behavior*.

[25] Lloyd-Richardson EE, Stanton CA, Papandonatos GD (2009). Motivation and patch treatment for HIV+ smokers: a randomized controlled trial. *Addiction*.

[26] Vidrine DJ, Marks RM, Arduino RC, Gritz ER (2012). Efficacy of cell phone-delivered smoking cessation counseling for persons living with HIV/AIDS: 3-month outcomes. *Nicotine and Tobacco Research*.

[27] Wewers ME, Neidig JL, Kihm KE (2000). The feasibility of a nurse-managed, peer-led tobacco cessation intervention among HIV-positive smokers. *The Journal of the Association of Nurses in AIDS Care*.

[28] Matthews AK, Conrad M, Kuhns L, Vargas M, King AC (2013). Project exhale: preliminary evaluation of a tailored smoking cessation treatment for HIV-positive African American smokers. *AIDS Patient Care and STDs*.

[29] Moadel AB, Bernstein SL, Mermelstein RJ, Arnsten JH, Dolce EH, Shuter J (2012). A randomized controlled trial of a tailored group smoking cessation intervention for HIV-infected smokers. *Journal of Acquired Immune Deficiency Syndromes*.

[30] Pedrol-Clotet E, Deig-Comerma E, Ribell-Bachs M, Vidal-Castell I, García-Rodríguez P, Soler A (2006). Bupropion use for smoking cessation in HIV-infected patients receiving antiretroviral therapy. *Enfermedades Infecciosas y Microbiologia Clinica*.

[31] Tornero C, Mafé C (2009). Varenicline and antiretroviral therapy in patients with HIV. *Journal of Acquired Immune Deficiency Syndromes*.

[32] Heatherton TF, Kozlowski LT, Frecker RC, Fagerstrom K-O (1991). The Fagerstrom test for nicotine dependence: a revision of the Fagerstrom Tolerance Questionnaire. *British Journal of Addiction*.

[33] DiClemente CC, Prochaska JO, Fairhurst SK, Velicer WF, Velasquez MM, Rossi JS (1991). The process of smoking cessation: an analysis of precontemplation, contemplation, and preparation stages of change. *Journal of Consulting and Clinical Psychology*.

[34] Fiore MC, Jaen CR, Baker TB (2008). *Treating Tobacco Use and Dependence: Clinical Practice Guideline*.

[35] Humfleet GL, Delucchi K, Kelley K, Hall SM, Dilley J, Harrison G (2009). Characteristics of HIV-positive cigarette smokers: a sample of smokers facing multiple challenges. *AIDS Education and Prevention*.

[36] Shuter J, Bernstein SL, Moadel AB (2012). Cigarette smoking behaviors and beliefs in persons living with HIV/AIDS. *The American Journal of Health Behavior*.

[37] Williams JM, Ziedonis D (2004). Addressing tobacco among individuals with a mental illness or an addiction. *Addictive Behaviors*.

[38] McCool RM, Richter KP (2003). Why do so many drug users smoke?. *Journal of Substance Abuse Treatment*.

[39] Prochaska JO, Velicer WF, Prochaska JM, Johnson JL (2004). Size, consistency, and stability of stage effects for smoking cessation. *Addictive Behaviors*.

[40] Sheeran P, Stroebe W, Hewstone M (2002). Intention—behavior relations: a conceptual and empirical review. *European Review of Social Psychology*.

[41] Chesney MA (2000). Factors affecting adherence to antiretroviral therapy. *Clinical Infectious Diseases*.

[42] Panel on Antiretroviral Guidelines for Adults and Adolescents Guidelines for the use of antiretroviral agents in HIV-1-infected adults and adolescents. http://aidsinfo.nih.gov/ContentFiles/AdultandAdolescentGL.pdf.

[43] Fiore MC, Baker TB (2011). Treating smokers in the health care setting. *The New England Journal of Medicine*.

[44] Crothers K, Goulet JL, Rodriguez-Barradas MC (2007). Decreased awareness of current smoking among health care providers of HIV-positive compared to HIV-negative veterans. *Journal of General Internal Medicine*.

[45] Horvath KJ, Eastman M, Prosser R, Goodroad B, Worthington L (2012). Addressing smoking during medical visits: patients with human immunodeficiency virus. *American Journal of Preventive Medicine*.

[46] Shuter J, Salmo LN, Shuter AD, Nivasch EC, Fazzari M, Moadel AB (2012). Provider beliefs and practices relating to tobacco use in patients living with HIV/AIDS: a national survey. *AIDS and Behavior*.

